# Identification and Functional Analysis of a Flavonol Synthase Gene from Grape Hyacinth

**DOI:** 10.3390/molecules24081579

**Published:** 2019-04-22

**Authors:** Hongli Liu, Beibei Su, Han Zhang, Jiaxin Gong, Boxiao Zhang, Yali Liu, Lingjuan Du

**Affiliations:** 1 College of Landscape Architecture and Arts, Northwest A&F University, Yangling 712100, Shaanxi, China; liuhongli1221@sina.com (H.L.); subeibei1106@163.com (B.S.); hange@nwafu.edu.cn (H.Z.); gongjiaxin@nwafu.edu.cn (J.G.); zhangboxiao@nwafu.edu.cn (B.Z.); 2 State Key Laboratory of Crop Stress Biology for Arid Areas, Northwest A&F University, Yangling 712100, Shaanxi, China; 3 Key Laboratory of Biology and Genetic Improvement of Horticultural Crops (Northwest Region), Ministry of Agriculture, Yangling 712100, Shaanxi, China

**Keywords:** copigmentation, flavonol, flower color, FLS, grape hyacinth

## Abstract

Flavonols are important copigments that affect flower petal coloration. Flavonol synthase (FLS) catalyzes the conversion of dihydroflavonols to flavonols. In this study, we identified a FLS gene, *MaFLS*, expressed in petals of the ornamental monocot *Muscari aucheri* (grape hyacinth) and analyzed its spatial and temporal expression patterns. qRT-PCR analysis showed that *MaFLS* was predominantly expressed in the early stages of flower development. We next analyzed the in planta functions of *MaFLS.* Heterologous expression of *MaFLS* in *Nicotiana tabacum* (tobacco) resulted in a reduction in pigmentation in the petals, substantially inhibiting the expression of endogenous tobacco genes involved in anthocyanin biosynthesis (i.e., *NtDFR*, *NtANS*, and *NtAN2*) and upregulating the expression of *NtFLS*. The total anthocyanin content in the petals of the transformed tobacco plants was dramatically reduced, whereas the total flavonol content was increased. Our study suggests that *MaFLS* plays a key role in flavonol biosynthesis and flower coloration in grape hyacinth. Moreover, *MaFLS* may represent a new potential gene for molecular breeding of flower color modification and provide a basis for analyzing the effects of copigmentation on flower coloration in grape hyacinth.

## 1. Introduction

Flower color is a key characteristic of ornamental plants [1]. Anthocyanins are flavonoids that contribute to the pink, red, orange, scarlet, violet, blue, and yellow pigmentation of ornamental plant flowers [2]. Copigmentation with other flavonoids influences the hue and intensity of flower color. Copigmentation can stabilize colored pigments and induce a blue shift in the final visible color [3]. Flavonols are one group of flavonoid that can act as copigments sandwiched between anthocyanins, causing color shifts and an increased color variety [1]. Furthermore, flavonols can themselves impart pale yellow or yellow coloration [4]. 

Both anthocyanins and flavonols are produced by branches of the flavonoid pathway and are derived from dihydroflavonols (dihydrokaempferol: DHK, dihydroquercetin: DHQ, and dihydromyricetin: DHM). Anthocyanins are synthesized by the sequential reaction of dihydroflavonol 4-reductase (DFR), anthocyanidin synthase (ANS), and glucosyltransferase (UFGT). In competition with DFR at a crucial branch point, individually acting FLS can catalyze the oxidation of dihydroflavonols to flavonols [4]. FLS is characterized as a soluble 2-ODD that requires cofactors 2-oxoglutarate, ferrous iron (II), and ascorbate and introduces the double bond between C-2 and C-3 of the C-ring [5,6]. Besides FLS, other 2-ODD enzymes, namely, flavanone 3β-hydroxylase (F3H), flavone synthase I (FNS I), and ANS, also function in flavonoid biosynthesis [7].

The first *FLS* cDNA was cloned from petunia (*Petunia hybrida*) and was functionally expressed in yeast and plants [6]. Additional *FLS* genes have since been identified and characterized in various plant species, such as *Arabidopsis thaliana* [8,9,10], *Eustoma grandiflorum* (lisianthus) [1], *Citrus unshiu* [11], *Glycine max* [12], *Gentiana triflora* [13], *Ginkgo biloba* [14], *Zea mays* (maize) [15], *Camellia nitidissima* [4], *Vaccinium corymbosum* [16], *Allium cepa* (onion) [17], *Cyclamen purpurascens* (cyclamen) [18], and *Litchi chinensis* [19]. There have been some reports of *FLS* genes in ornamental plants that function in flower coloration. Holton et al. demonstrated that antisense expression of a *FLS* gene in petunia reduced flavonol synthesis and changed the light pink coloration of petals and filaments to red. Similarly, constitutive overexpression of a *FLS* gene from lisianthus produced flowers with a deeper magenta coloration than the wild type (WT) plants [1]. Heterologous expression of *CnFLS1* in *Nicotiana tabacum* (tobacco) changed floral coloration, resulting in white flowers [4]. Akita et al. isolated two *FLS* genes (*CpurFLS1* and *CpurFLS2*) and discussed their involvement in flower coloration in cyclamen [18]. Furthermore, previous studies also showed that FLS can cooperate with DFR to control the metabolic balance between the anthocyanin and flavonol branches of the flavonoid pathway to ultimately determine the formation of flower color [20], or identified the transcription factor R2R3-MYB, which regulates FLS to affect spatial pattern variation of floral pigments [21].

Grape hyacinth (*Muscari* spp.), named for its unique grape-like color and shape, is a monocotyledonous ornamental bulbous plant that blossoms in mid-spring [22,23]. Generally, grape hyacinth varieties display a single color, such as white, pink, purple, azure, cobalt, violet, or lavender. Rarely, some species produce upper flowers that are a different color to the lower flowers [22]. Because of its variable flower coloration, grape hyacinth is a good model to study the secondary metabolism of flower coloration in monocots [22,24]. Previous studies of grape hyacinth flower coloration have focused on the chemical basis of anthocyanin-related pigmentation and on the functions of structural and regulatory proteins involved in anthocyanin biosynthesis [22,23,25]. Few studies have examined the compounds involved in copigmentation or analyzed the genes affecting copigmentation. It is valuable to elucidate the mechanism of flower color formation or create the novel color morphs of grape hyacinth via molecular regulation of the key gene (*FLS*) for flavonol synthesis.

In this study, we identified a *FLS* gene expressed in grape hyacinth petals, *MaFLS*, and analyzed its spatial and temporal expression patterns. Moreover, we measured flavonol contents at different floral developmental stages and in different organs. Via heterologous expression in tobacco, we examined the function of *MaFLS* in planta by examining the effect on phenotype, flavonoid content, and expression of endogenous genes in the petals of the transgenic tobacco lines. We discuss the potential role of *MaFLS* during floral coloration of grape hyacinth. Overall, our study provides further evidence to a growing literature on FLS–DFR competition and provides the basis for analyzing the effects of copigmentation on flower coloration in grape hyacinth.

## 2. Results

### 2.1. MaFLS Gene Cloning and Sequence Analysis

We searched for FLS homologs in the transcriptome of *M. armeniacum* flowers [24] by local BlastP querying with the characterized *A. thaliana FLS1* [9,26,27]. One FLS homolog was identified and designated *MaFLS* (GenBank accession number MH636605) and its cDNA was isolated by RACE-PCR and PCR. *MaFLS* consisted of 1418 nucleotides with a poly(A) tail, of which 993 bp represented an ORF encoding 330 amino acid residues, with a molecular weight of 36.45 kDa and a theoretical pI of 6.33. 

**Figure 1:**
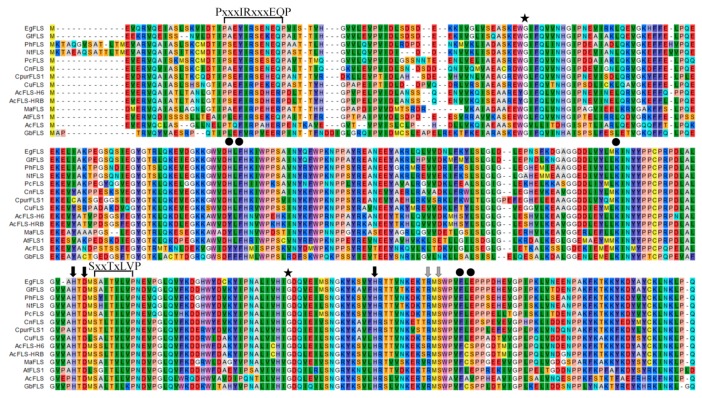
Multiple alignment of the predicted amino acid sequence of MaFLS with that of other flavonol synthase (FLS) proteins. MaFLS is compared with FLS sequences from *Eustoma grandiflorum* (EgFLS), *Gentiana triflora* (GtFLS), *Petunia hybrida* (PhFLS), *Nicotiana tabacum* (NtFLS), *Petroselinum crispum* (PcFLS), *Camellia nitidissima* (CnFLS), *Cyclamen purpurascens* (CpurFLS1), *Citrus unshiu* (CuFLS), *Allium cepa* (AcFLS-H6 and AcFLS-HRB), *Arabidopsis thaliana* (AtFLS1), *Acacia confusa* (AcFLS), and *Ginkgo biloba* (GbFLS). The alignment was generated using CLC sequence viewer 8.0. The FLS-specific motifs “PxxxIRxxxEQP” and “SxxTxLVP” are indicated. Black arrows indicate the ferrous iron-binding residues (His216, Asp218, and His272). Gray arrows represent the 2-oxoglutarate binding residues (Arg282 and Ser284). Black dots show the putative DHQ-binding residues (Tyr127, Phe129, Lys197, Phe288, and Ser290). Asterisks indicate the residues responsible for the proper folding of the FLS polypeptide (Gly65 and Gly256).

Comparison of the deduced MaFLS amino acid sequence and that of other FLS proteins with known functions revealed that it shared 72% and 71% identity with monocot *Allium cepa* AcFLS-HRB (AY647262) and AcFLS-H6 (AY221247), respectively; 67% identity with *Citrus unshiu* CuFLS (AB011796); 64% identity with *Gentiana triflora* GtFLS (AB587658); and 63% identity with *Camellia nitidissima* CnFLS (ADZ28516). Moreover, MaFLS harbored characteristic conserved ferrous iron-binding residues (His216, Asp218, and His272; marked by black arrows; Figure 1) and the 2-oxoglutarate binding residues (Arg282 and Ser284; marked by gray arrows; Figure 1) of FLS proteins. This clearly indicated that MaFLS belonged to the soluble Fe^2+^/2-ODD protein family. Furthermore, MaFLS possessed five key amino acids (Tyr127, Phe129, Lys197, Phe288, and Ser290; marked by black dots; Figure 1) that were identified as potential active DHQ binding site residues, highly conserved in FLSs of various species [26]. In addition, MaFLS contained the FLS-specific motifs “PxxxIRxxxEQP” and “SxxTxLVP”, which can be used to distinguish FLSs from other plant 2-ODDs, like F3H, ANS, and FNS. MaFLS possessed the residues responsible for the proper folding of the FLS polypeptide (Gly65 and Gly256; marked by asterisks; Figure 1), which are conserved in all 2-ODD proteins [17]. 

**Figure 2:**
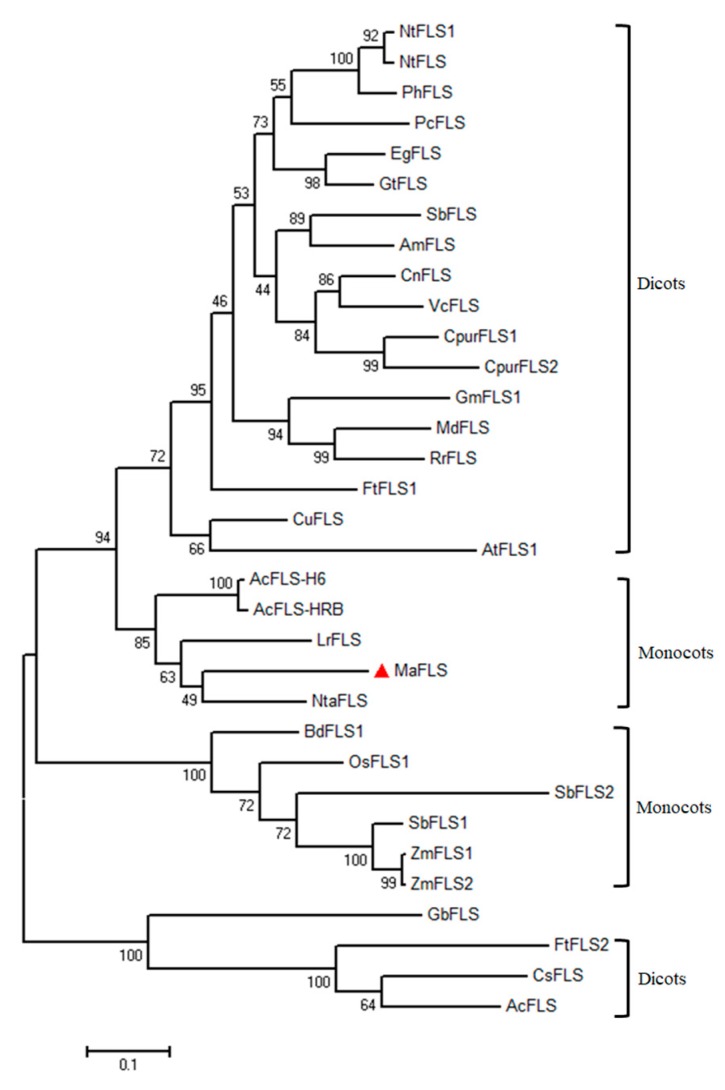
Phylogenetic analysis of MaFLS and other FLS proteins. The maximum-likelihood phylogenetic tree was generated using MEGA 6.0 software. Numbers at each interior branch indicate the bootstrap values of 1000 replicates. The bar indicates a genetic distance of 0.1. GenBank accession numbers are as follows: *Muscari aucheri* MaFLS (MH636605, marked with red triangle), *Nicotiana tabacum* NtFLS1 (ABE28017) and NtFLS (AB289451), *Petunia hybrida* PhFLS (CAA80264), *Petroselinum crispum* PcFLS (AAP57395), *Eustoma grandiflorum* EgFLS (AAF64168), *Gentiana triflora* GtFLS (AB587658), *Scutellaria baicalensis* SbFLS (KC404852), *Antirrhinum majus* AmFLS (ABB53382), *Camellia nitidissima* CnFLS (ADZ28516), *Vaccinium corymbosum* VcFLS (KP334105), *Cyclamen purpurascens* CpurFLS1(LC210072) and CpurFLS2 (LC210073), *Glycine max* GmFLS1 (AB246668), *Malus domestica* MdFLS (AY965343), *Rosa rugosa* RrFLS (KM099095), *Fagopyrum tataricum* FtFLS1 (JF274262) and FtFLS2 (JX401285), *Citrus unshiu* CuFLS (AB011796), *Arabidopsis thaliana* AtFLS1 (AAB41504), *Allium cepa* AcFLS-H6 (AY221247) and AcFLS-HRB (AY647262), *Lilium regale* LrFLS(ASV46329), *Narcissus tazetta* NtaFLS (AFS63900), *Brachypodium distachyon* BdFLS1 (XM_003570514), *Oryza sativa* OsFLS1 (NP_001048230), *Sorghum bicolor* SbFLS1 (XP_002454608) and SbFLS2 (EES00073), *Zea mays* ZmFLS1(BT039956) and ZmFLS2 (XP_008646309), *Ginkgo biloba* GbFLS (ACY00393), *Camellia sinensis* CsFLS (ABM88786), and *Acacia confusa* AcFLS (JN812062).

To determine the relationship between the putative MaFLS protein and other plant FLSs, we performed a phylogenetic analysis with the functionally characterized plant FLSs as well as various putative FLSs (Figure 2). MaFLS was grouped with fellow monocots close to *Narcissus tazetta* NtaFLS (AFS63900), *Allium cepa* AcFLS-H6 (AY221247), and AcFLS-HRB (AY647262) in the order Asparagales, and *Lilium regale* LrFLS (ASV46329) in the order Liliales (Figure 2). MaFLS showed a marked separation from enzymes of dicot plants and of monocot Poaceae grasses. These results indicate that the phylogenetic analysis fits well with the genetic relationships among the species.

### 2.2. Correlation Analysis of MaFLS Expression Levels and Total Flavonol Content

**Figure 3:**
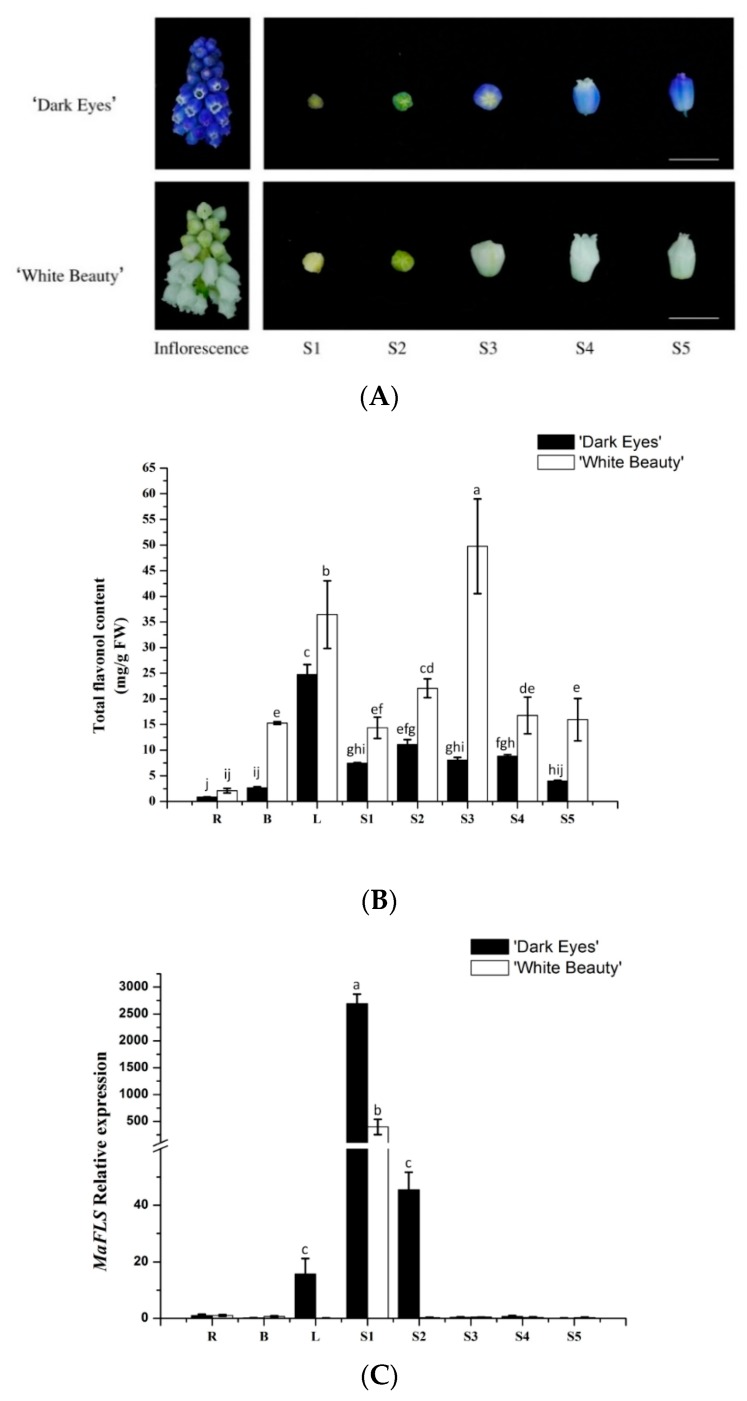
The flavonol content and expression profiles of *MaFLS* in *M. aucheri* “Dark eyes” and *M. aucheri* “White Beauty”. (**A**) The inflorescence and the petals at five flower developmental stages of *M. aucheri* “Dark eyes” and *M. aucheri* “White Beauty”. Bars, 5 mm. (**B**) The total flavonol content of petals. FW: Fresh weight. (**C**) The expression profile of *MaFLS.* Flavonol content and *MaFLS* expression were determined in roots (R), bulbs (B), leaves (L), and petals at five flower developmental stages (S1–S5). *MaActin* was used as the internal expression control. Each column represents means ± SD from three technical replicates. Different letters above the bars indicate significantly different values (*P* < 0.05) calculated using one-way ANOVA followed by a Duncan’s multiple range test.

We extracted metabolites from roots, bulbs, leaves, and petals at the five floral developmental stages (S1–S5) of “White Beauty” and “Dark Eyes” (Figure 3A). HPLC analysis revealed that total flavonol content in all organs of “White Beauty” was significantly higher than that of “Dark Eyes”. In “White Beauty”, total flavonol content increased during flower development, peaked before blooming, and then decreased gradually after petal expansion (Figure 3B). However, in “Dark Eyes”, the total flavonol content was higher in S2 buds than in the buds at other developmental stages. In addition, total flavonols also accumulated in other vegetative organs, especially in leaves of the two grape hyacinth cultivars. Recently, Lou et al. reported that the level of total anthocyanins in violet-blue flowers such as those of “Dark Eyes” was substantially higher than that in the white flowers of “White Beauty” [22]. Therefore, these results likely reflect relatively higher flavonol accumulation and extremely low anthocyanin accumulation during the formation of white flowers in “White Beauty”. 

Furthermore, we determined the expression levels of *MaFLS* in different organs and flower development stages by qRT-PCR analysis. *MaFLS* transcript levels were highest in S1 buds, whereas *MaFLS* expression was undetectable in S2–5 petals and in the roots, bulbs, and leaves of *M. aucheri* “White Beauty” (Figure 3C). Similarly, in *M. aucheri* “Dark eyes”, *MaFLS* expression was extraordinarily high in S1 buds, markedly lower in S2 petals, and undetectable in S3–5 petals. Moreover, *MaFLS* expression was barely detectable in the roots and bulbs, and slightly detectable in the leaves of *M. aucheri* “Dark eyes” (Figure 3C). Thus, *MaFLS* is predominantly expressed in the early stages of flower development in grape hyacinth, which is consistent with reports in other ornamental plants, such as carnation (*Dianthus caryophyllus*), petunia, lisianthus, and cyclamen [1,6,18,28]. Together, our results show that *MaFLS* likely plays a key role in flower coloration, alongside other copigmentation-related *FLS* genes. Nevertheless, *MaFLS* expression is not concomitant with the accumulation of total flavonols in grape hyacinth. 

### 2.3. In Vivo Localization of MaFLS

**Figure 4:**
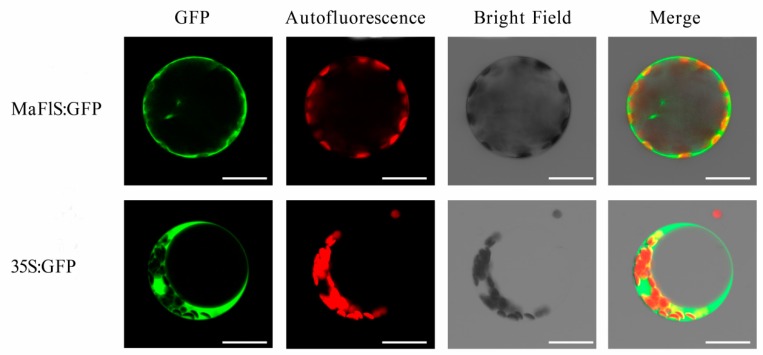
Subcellular localization of MaFLS proteins. Transient expression of the MaFLS-GFP fusion protein and the 35S:GFP control in *A. thaliana* mesophyll protoplasts. GFP, GFP fluorescence; autofluorescence, chloroplast autofluorescence; merge is an overlay of chloroplast autofluorescence, GFP fluorescence, and bright-field images. Bars, 25 mm.

To detect the subcellular localization of MaFLS, the positive control 35S:GFP (pBI221) and the recombinant plasmid pBI221-MaFLS-GFP were transformed into *A. thaliana* mesophyll protoplasts. As shown in Figure 4, fluorescence from 35S:GFP was dispersed throughout *A. thaliana* mesophyll protoplasts, whereas fluorescence from the MaFLS:GFP fusion protein was mainly localized in the cytosol and cell periphery. Therefore, MaFLS is both a cytoplasmic and cell periphery protein. 

### 2.4. Heterologous Expression of MaFLS in Tobacco Alters Petal Color

**Figure 5:**
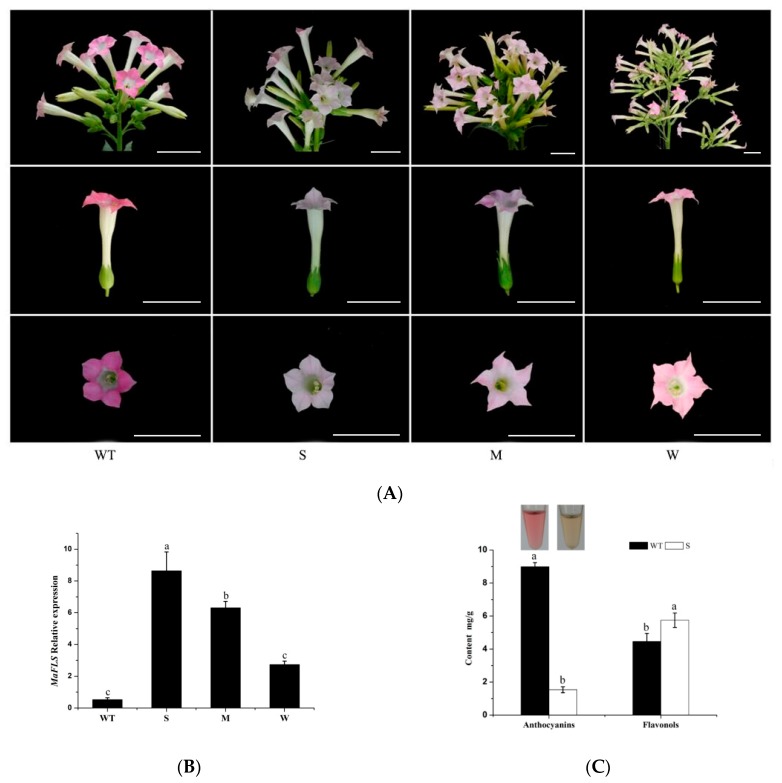
Changes in tobacco flowers induced by heterologous expression of *MaFLS.* (**A**) Heterologous expression of *MaFLS* in transgenic tobacco flowers resulted in a clear phenotypic change in petal coloration. Shown are non-transformed controls (WT) and three *MaFLS*-expressing lines exhibiting different phenotypic characteristics (S, strong; M, medium; W, weak). Bars, 5 cm. (**B**) qRT-PCR analysis of *MaFLS* relative expression levels in the petals of three independent transgenic tobacco lines. *NtTubA1* was used as the internal expression control. (**C**) The color of the extractions is shown in the centrifugal tubes. HPLC analysis of anthocyanin and flavonol levels in tobacco petals in mg/g fresh weight (FW) of the non-transformed control (wild type, WT) and the transgenic line with a strong phenotype (S). Each column represents means ± SD from three technical replicates. Different letters above the bars indicate significantly different values (*P* < 0.05) calculated using one-way ANOVA followed by Duncan’s multiple range test.

To ascertain the function of MaFLS in flower coloration, *MaFLS* was transformed into tobacco (*N. tabacum* “NC89”) under the control of the 35S-CaMV promoter for heterologous expression experiments. We identified 11 transformant lines by PCR analysis, using the vector-specific primers 2300-F and 2300-R (Table S1). Compared with the deep pink flowers of wild-type tobacco, the transgenic tobacco lines heterologously expressing *MaFLS* showed reduced levels of petal pigmentation (Figure 5A). Five lines displayed a severe phenotype (S) of pale pink to completely white petals; three lines displayed a medium phenotype (M) of pink petals, whereas the remaining lines displayed a weak phenotype (W) of pale pink petals (Figure 5A). 

To further investigate *MaFLS* expression levels in the transgenic tobacco lines, we carried out qRT-PCR analysis using *MaFLS*-specific qRT-PCR primers (Table S1). We discovered that the extent of change in petal coloration was consistent with the relative expression levels of *MaFLS* in the transgenic tobacco lines, in that the lines with a severe and a weak phenotype had the highest and lowest *MaFLS* expression levels, respectively (Figure 5B). Therefore, the changes in petal color in the transgenic tobacco lines appear to be the result of heterologous *MaFLS* expression. 

In addition, we further investigated the changes in anthocyanin and flavonol levels in petals from transgenic tobacco lines with a severe phenotype by HPLC. The color of the extractions indicated that the petals of lines with a severe phenotype contained less anthocyanins than the non-transformed control (Figure 5C). This was confirmed by HPLC analysis, which showed that the severe phenotype lines had a clear reduction in total anthocyanin levels; however, they accumulated higher levels of total flavonols, compared to the non-transformed control (Figure 5C). These results strongly suggest that high-level *MaFLS* expression in transgenic tobacco lines causes a remarkable decrease in anthocyanin content and an increase in flavonol accumulation in vivo, which results in a severe reduction in petal pigmentation.

### 2.5. Heterologous Expression of MaFLS in Tobacco Affects the Expression of Anthocyanin Pathway Genes

**Figure 6:**
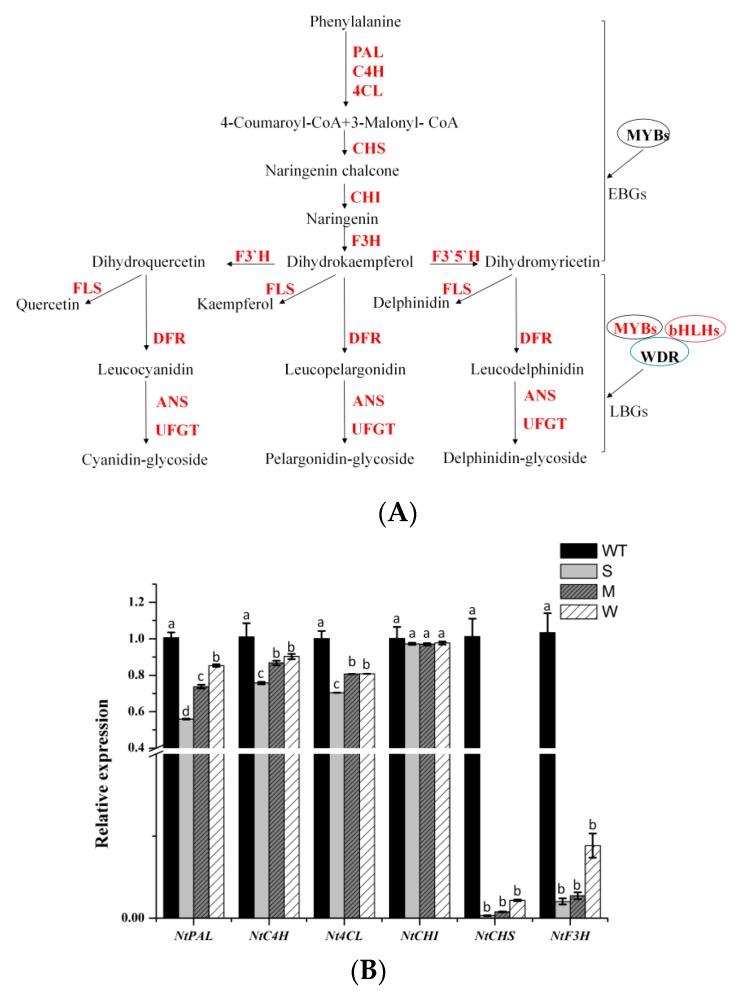

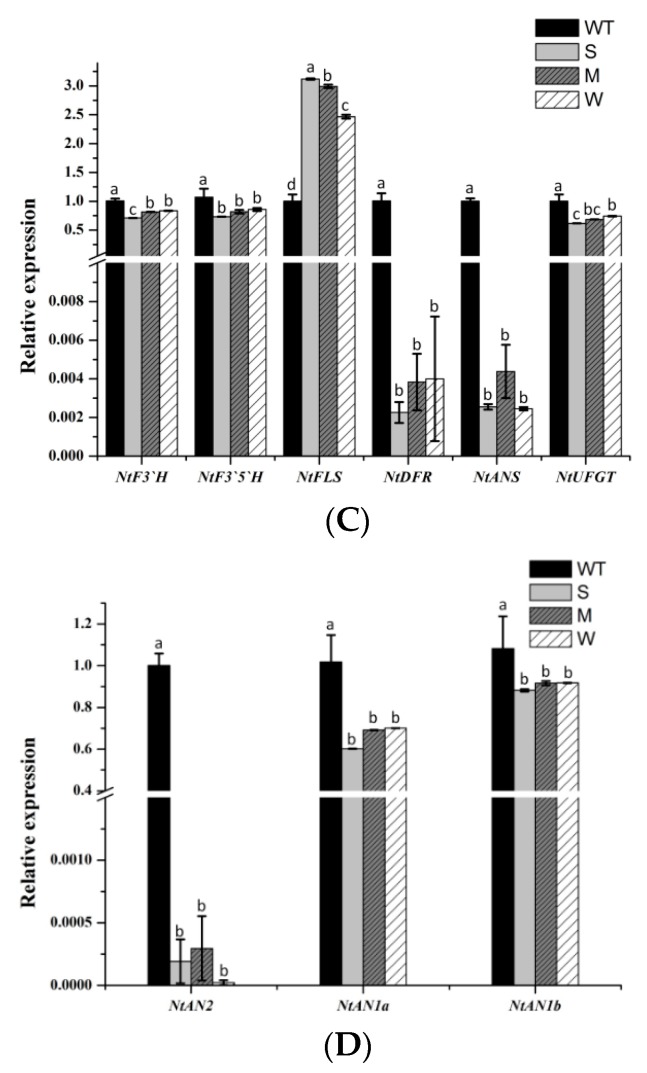
A schematic diagram of flavonoid biosynthetic pathway and expression profiles of anthocyanin biosynthetic endogenous TF genes in petals. (**A**) A schematic diagram of flavonoid biosynthetic pathway. EBGs, the early biosynthetic genes; LBGs, the late biosynthetic genes; PAL, phenylalanine ammonia-lyase; C4H, cinnamate 4-hydroxylase; 4CL, 4-coumarate: CoA ligase; CHS, chalcone synthase; CHI, chalcone isomerase; F3H, flavanone 3-hydroxylase; F3’H, flavonoid 3’-hydroxylase; F3’5’H, flavonoid 3’5’-hydroxylase; FLS, flavonol synthase; DFR, dihydroflavonol 4-reductase; ANS, anthocyanidin synthase; UFGT, uridine diphosphate-sugar: Flavonoid glycosyltransferases; MYBs, MYB transcription factors; bHLHs, basic helix-loop-helix proteins; WDR, WD repeat protein. The analyzed genes are highlighted in red. (**B**)The expression patterns of *NtPAL*, *NtC4H*, *Nt4CL*, *NtCHS*, *NtCHI*, and *F3H* in petals. (**C**) The expression patterns of *NtF3’H*, *NtF3’5’H*, *NtFLS*, *NtDFR*, *NtANS*, and *NtUFGT* in petals. (**D**) The expression patterns of three regulatory genes (*NtAN2*, *NtAN1a*, and *NtAN1b*) in petals. Data are for the non-transformed control (WT) and three *MaFLS*-expressing lines exhibiting different phenotypic characteristics (S, strong; M, medium; W, weak). Each column represents means ± SD from three technical replicates. Different letters above the bars indicate significantly different values (*P* < 0.05) calculated using one-way ANOVA followed by Duncan’s multiple range test.

Subsequently, we conducted a qRT-PCR expression analysis of endogenous genes involved in the flavonoid biosynthetic pathway in the petals of *MaFLS*-expressing transgenic tobacco (Figure 6A, primers are listed in Table S1). The analyzed genes included twelve structural genes (*NtPAL*, *NtC4H*, *Nt4CL*, *NtCHS*, *NtCHI*, *NtF3H*, *NtF3’H*, *NtF3’5’H*, *NtFLS*, *NtDFR*, *NtANS*, and *NtUFGT*) and three regulatory genes (*NtAN2*, *NtAN1a*, and *NtAN1b*), which are highlighted in red (Figure 6A). 

Based on the one-way ANOVA statistical results presented in Figure 6, only the expression level of *NtCHI* was not significantly different between the *MaFLS*-expressing lines and the non-transformed control; all the other genes showed the decreased expressions. In particular, *NtCHS*, *NtF3H*, *NtDFR*, *NtANS*, and *NtAN2* expression was remarkably lower in the *MaFLS*-expressing lines than in the non-transformed control (Figure 6B–D). Particularly, the transcript levels of two endogenous genes (*NtDFR* and *NtANS*) and one regulatory gene (*NtAN2*), closely related to anthocyanin biosynthesis, showed remarkable reductions in three *MaFLS*-expressing lines, compared with the non-transformed control (Figure 6C,D). Conversely, the expression of endogenous *NtFLS* was markedly upregulated in the *MaFLS*-expressing lines. These results suggest that heterologous expression of *MaFLS* in tobacco petals substantially inhibited expression of the key structural genes (*NtDFR* and *NtANS*) and the vital regulatory gene (*NtAN2*) that have been implicated in anthocyanin biosynthesis and upregulated the expression of endogenous *NtFLS*.

## 3. Discussion

Anthocyanins and flavonols are important flavonoid phytochemicals that contribute to flower coloration. Understanding the molecular and biochemical controls of anthocyanin and flavonol pathways, or studying related genes, is an important focus in the ornamental plant industry, since novel color morphs can be profitable. Here, we describe the cloning and molecular characterization of *MaFLS*, the first FLS characterized from ornamental monocots. Sequence alignments revealed that MaFLS possesses the HxDxnH and RxS motifs for binding ferrous iron and 2-OG, respectively. Moreover, MaFLS has the five amino acid residues (Tyr127, Phe129, Lys197, Phe288, and Ser290; Figure 1) responsible for substrate binding [26]. It is noteworthy that Tyr127 and Ser290 are not conserved. Similar to Tyr132 in AcFLSs and Phe137 in GbFLS [14,17], the presence of Tyr127 in MaFLS (instead of the His, residue commonly present in other FLSs at this position) might impart improved enzyme catalytic activity for quercetin production [26]. Because the previous study showed that it is a potentially attractive substrate binding site for modification to engineer a FLS with improved activity [26].

Some progress has recently been made in analyzing the anthocyanin profiles of *Muscari* [22,24,29,30,31]. However, few reports have focused on the flavonol profiles in *Muscari*. Our previous work revealed that flavonols in grape hyacinth petals are mainly derivatives of kaempferol and quercetin, in accordance with flavonol accumulation in lisianthus petals [32]. Here, we determined the levels of total flavonols in two grape hyacinth cultivars, one with white flowers and the other with blue. We found that total flavonol content of the white-flower cultivar “White Beauty” was significantly higher than that of blue-flower cultivar “Dark Eyes”. How flavonols regulate flower color in these cultivars is unclear, but one possibility is that they act as copigments that participate in petal coloration.

In addition, we observed little correlation between the expression levels of *MaFLS* and the accumulation of total flavonols in grape hyacinth. Conversely, positive correlations between flavonoid concentration and mRNA levels of *FLS* genes have been reported in other plant species [19]. First, this correlation depends on the availability of primary substrate (dihydroflavonols) flux in the pathway, because only some of them can be catalyzed by MaFLS to the corresponding flavonols, while others may synthesize many other molecules that were not measured in this analysis. Second, this phenomenon perhaps indicates that other *MaFLS* isogenes may be expressed to produce the appropriate flavonols in grape hyacinth, as is the case in *A. thaliana*, lisianthus, and onion [1,9,17]. However, only one putative *MaFLS* sequence has been identified thus far. Due to a lack of sufficient genomic resources, we are unaware of other *FLS* genes that exist in grape hyacinth. Nevertheless, we speculate that the *MaFLS* characterized here promotes flavonol production to act as UV-B sunscreens and protect young developing floral tissue and that these colorless flavonols may act as copigments sandwiched between anthocyanin molecules to induce a blue shift [1,12]. Furthermore, these colorless flavonols might act as UV-spectrum flower pigments, contributing to the attractive and defensive functions for insects [33], or they might be important for male fertility and auxin metabolism and transport [34,35].

At the subcellular level, MaFLS appears to localize to the cytoplasm and cell periphery, but not to the nucleus, in accordance with the observation that flavonoids are synthesized and localized to the cytoplasm in Arabidopsis [36]. However, there are many flavonoid metabolic enzymes for which dual cytoplasmic/nuclear localization has been observed, such as CHS, CHI, and FLS [35]. 

We have shown that constitutive heterologous expression of *MaFLS* influenced flower color in transgenic tobacco lines. HPLC analysis showed a dramatic decrease in the levels of total anthocyanins and an increase in the levels of total flavonols in the resulting tobacco flowers. The severity of the flower color phenotype was highly consistent with the *MaFLS* transgene expression level. It is noteworthy that over-expression of *MaFLS* in tobacco inhibited expression of the genes involved in the early step of flavonoid biosynthesis, particularly dampening the expression of the key structural genes (*NtDFR* and *NtANS*) and the vital regulatory gene (*NtAN2*) in the anthocyanin synthesis pathway. This phenomenon may be explained as a feedback mechanism existing in the flavonoid pathway that affected the expression of genes in the early step of flavonoid biosynthesis [20]. The result also further demonstrated that the competition between FLS and DFR genes ultimately determines the flower coloration. 

## 4. Materials and Methods

### 4.1. Plant Materials and Growth Conditions

Grape hyacinth (*M. aucheri* “White Beauty” and *M. aucheri* “Dark Eyes”) plants were grown in the experimental field of the Northwest A&F University at Yangling Distruct in Shaanxi Province, China. The petals of grape hyacinth were divided into five floral development stages (S1–S5), according to the previous description (Figure 3A) [22]. To obtain RNA and metabolite samples, healthy tissues and flowers were collected, frozen immediately in liquid nitrogen, and stored at −80 °C. Tobacco plants (*Nicotiana tabacum* “NC89”) for transformation were grown aseptically from seed on Murashige and Skoog medium, supplemented with 3% (*w*/*v*) sucrose, and transgenic tobacco flowers were harvested at the full-bloom stage.

### 4.2. Cloning of Full-Length MaFLS cDNAs

A *FLS* homolog was identified in the transcriptome of grape hyacinth [24], designated *MaFLS*. To obtain the full-length cDNA sequence of *MaFLS*, we performed both 5′- and 3′-RACE experiments, using the flowers of *M. aucheri* “White Beauty”, as in the previous method [23]. The primers used for 501 and 3′-RACE PCR and full-length gene cloning are listed in Table S1. The obtained *MaFLS* cDNA sequence was submitted to the NCBI GenBank database (accession number MH636605).

### 4.3. Sequence Alignment and Phylogenetic Analysis

Full-length amino acid sequences of MaFLS and other FLS proteins were retrieved from the GenBank database. Multiple sequence alignments were performed with CLC sequence viewer 8.0 software. FLS-specific motifs and conserved amino acid residues were indicated by different colored symbols. The phylogenetic tree was constructed by the maximum likelihood method with 1000 bootstrap replicates using MEGA 6.0. 

### 4.4. RNA Isolation and qRT-PCR Analysis

Total RNA was extracted from frozen tissue of the flowers, roots, bulbs, and leaves of grape hyacinth (*M. aucheri* “White Beauty” and *M. aucheri* “Dark eyes”) as well as flowers of tobacco (NC89), using the Omega Total RNA Kit (Omega, Norcross, GA, USA). Purified RNA was assessed using agarose electrophoresis and measured on a Nanodrop 2000 (Thermo Scientific). Then, 1-μg RNA aliquots were used for reverse transcription to cDNA, using the PrimeScript™ RT Reagent Kit (TaKaRa Biotechnology, Dalian, China). The cDNA was diluted five-fold and used as the template for qRT-PCR. NovoStart^®^SYBR qPCR SuperMix Plus (Novoprotein, Shanghai, China) was used as the fluorochrome for the qRT-PCR assay. The assay was conducted using the iQ5 RT-PCR detection system (Bio-Rad, Hercules, CA, USA). Each reaction mixture consisted of NovoStart^®^SYBR qPCR SuperMix Plus with 0.8 μL of forward and reverse primers each, 1 μL of cDNA, and 7.4 μL of ddH_2_O in a final volume of 20 μL. The amplification protocol was 95 °C for 1 min, followed by 40 cycles of 95 °C for 20 s, 58 to 62 °C for 20 s, and 72 °C for 30 s. The qRT-PCR primers of grape hyacinth and tobacco are listed in Table S1. *MaActin* and *NtTubA1* were used as the internal control genes in each grape hyacinth and tobacco sample, respectively. All analyses were conducted in technical triplicate. 

### 4.5. Subcellular Localization of MaFLS

To investigate the subcellular localization of MaFLS, the ORF of *MaFLS* without a termination codon was inserted between the *Xba*I and *Kpn*I sites of the pBI221-GFP vector, using the Seamless Cloning and Assembly Kit (Novoprotein, China; primers are listed in Table S1) to generate the recombinant plasmid pBI221-MaFLS-GFP. Using the PEG-calcium mediated transfection method [37], the recombinant plasmid pBI221-MaFLS-GFP was transformed into *A. thaliana* mesophyll protoplasts. After 16–18 h of cultivation, the transformed protoplasts were observed with a confocal laser scanning microscope (TCS SP8, Leica, Wetzlar, Germany).

### 4.6. Heterologous Expression Vector Construction and Stable Tobacco Transformation

For constructing the *MaFLS* heterologous expression vector, the *MaFLS* ORF sequence without a termination codon was inserted between *Kpn*I and *Xba*I of the pCAMBIA2300 vector, using the Seamless Cloning and Assembly Kit (Novoprotein, China; primers are listed in Table S1) to produce the recombinant plasmid p2300-MaFLS. Then, the recombinant plasmid p2300-MaFLS was introduced into *Agrobacterium tumefaciens* Gv3101 (MP) by electroporation. Tobacco leaf disk transformation was conducted using a previously described protocol [38]. T0 generation of transgenic tobacco lines heterologously expressing MaFLS were used for qPCR analysis, and the lines showing a severe phenotypic change in petal pigmentation were used for further HPLC analysis. 

### 4.7. HPLC Analysis 

Petals from the five floral developmental stages; roots, bulbs, and leaves of grape hyacinth; and the fresh petals of transgenic tobacco were ground to powder in liquid nitrogen and extracted with methanol to H_2_O to formic acid to trifluoroacetic acid (70:27:2:1, *v*/*v*/*v*/*v*) for analysis of total anthocyanins [39,40] or extracted with methanol for analysis of total flavonols [41]. The supernatant was filtered through 0.22-μm Millipore filters. Total anthocyanin content of tobacco flowers was measured using a UV-visible spectrophotometer (UV2600, Shimadzu, Kyoto, Japan) and calculated according to the equation: QAnthocyanins = (A530 − 0.25 × A657) × M^−1^. 

Flavonol was detected at 360 nm using reverse HPLC analysis, as in the previous descriptions [22,23]. The results were expressed as milligrams of anthocyanins or flavonols per gram of fresh weight (mg/g FW). All samples were analyzed in three biological replicates.

### 4.8. Statistical Analysis 

Analysis of variance (one-way ANOVA) was performed using the SAS program (version 8.0, SAS Institute, Cary, NC, USA). The statistical difference was compared by the Duncan’s multiple range test (*P* < 0.05 was considered significant).

## 5. Conclusions

In this study, we identified a *FLS* gene, *MaFLS*, which is predominantly expressed in the early stages of flower development in grape hyacinth. Heterologous expression of *MaFLS* in tobacco showed a reduction in pigmentation in the petals because of a remarkable decrease in anthocyanin content and an increase in flavonol accumulation. Moreover, our study demonstrates the vital role of *MaFLS*, to promote flavonol production in young developing floral tissue, and that these flavonols may play a key role in flower coloration in grape hyacinth. However, MaFLS-based regulation does not seem to fully explain the flower coloration of grape hyacinth. Future research should be conducted to determine the following: (1) How *MaFLS* and *MaDFR* gene products compete for common substrates to regulate flavonoid biosynthesis and coloration, (2) how flavonol and anthocyanin biosynthesis is regulated under the control of transcriptional regulators, and (3) how the precise ratio of flavonol and anthocyanin metabolites affects flower coloration.

## Supplementary Materials

The following are available online.

## Abbreviations

FLS	flavonol synthase.
DFR	dihydroflavonol 4-reductase
ANS	anthocyanidin synthase
qRT-PCR	quantitative real-time PCR
DHK	dihydrokaempferol
DHQ	dihydroquercetin
ORF	open reading frame
PCR	polymerase chain reaction
UFGT	glucosyltransferase
2-ODD	2-oxoglutarate-dependent dioxygenase
