# Supplementary material for: Identification and Functional Analysis of a Flavonol Synthase Gene from Grape Hyacinth
Source: Molecules. 2019 Apr 22;24(8):1579. doi: 10.3390/molecules24081579 (PMC6514955; doi:10.3390/molecules24081579)
Supplement: Supplementary file 1 [file molecules-24-01579-s001.pdf]

Table S1. Primers used in this study.

Primer	Sequence (5′–3′)
MaFLS-5′RACE	GCCCTAACCCTAGCGACAGGCTGCCCAGC
MaFLS-3′RACE	CCAGCTGGTGAACCACGGGATACCCGCCG
MaFLS-full length cDNA-F	ATGGACATGGAGAGGGTGCAAGC
MaFLS-full length cDNA-R	TCACTGCGGAAGCTTGTTGATCTTGC
MaFLS-F-2300-KpnI	ACGGGGGACGAGCTCGGTACCATGGACATGGAGAGGGTGCAAGC
MaFLS-R-2300-XbaI	CACCATGGTGTCGACTCTAGACTGCGGAAGCTTGTTGATCTTGCAG
MaFLS-F-221-XbaI	GAGAACACGGGGGACTCTAGAATGGACATGGAGAGGGTGCAAG
MaFLS-R-221-KpnI	TTCTCCCTTACCCATGGTACCCTGCGGAAGCTTGTTGATCT
2300-F	GGAAGGTGGCTCCTACAAATGC
2300-R	CTGCTTCATGTGGTCGGGGTAG
MaFLS-qPCR-F	GCAGGAGGAGAAGGAGTCTTA
MaFLS-qPCR-R	CCAGACGTTGTGGAACAAGTA
MaActin-qPCR-F	AACATTCAGAAAGAGTCCACCC
MaActin-qPCR-R	GCTTACCAGCAAAGATCAACCG
NtPAL-qPCR-F	AGCTAGTAGTGATTGGGTTATGG
NtPAL-qPCR-R	CCGTTCTTGGTTCTCCTATGT
NtC4H-qPCR-F	GAGAAGCACGTTGAGGCTAAT
NtC4H-qPCR-R	GTCCCAAGGTAATGCCAAGAA
Nt4CL-qPCR-F	TGTTGACGCACAAGGGATTA
Nt4CL-qPCR-R	CAAAGGCAACACACACATCAA
NtCHS-qPCR-F	TGACACCCACTTGGATAGTTTAG
NtCHS-qPCR-R	CGACCTCTGGAATTGGATCAG
NtCHI-qPCR-F	CTTTTCTCGCCGCTAAATG
NtCHI-qPCR-R	TTTCTGCCACCTTCTCTG
NtF3H-qPCR-F	CAAGGCATGTGTGGATATGG
NtF3H-qPCR-R	TGTGTCGTTTCAGTCCAAGG
NtF3′H-qPCR-F	AGGCTCAACACTTCTCGT
NtF3′H-qPCR-R	CATCAACTTTGGGCTTCT
NtF3′ 5′H-qPCR-F	CGCACTACCATACTTAGGAGCCAT
NtF3′ 5′H-qPCR-R	CAGCATCAGGAGTAGAAGCAACAG
NtFLS-qPCR-F	GGTTAGGAGCCCATGAAATGA
NtFLS-qPCR-F	CACAACACCAAGTGCCAAATC
NtDFR-qPCR -F	AACCAACAGTCAGGGGAATG
NtDFR-qPCR –R	TTGGACATCGACAGTTCCAG

NtANS-qPCR -F	TGGCGTTGAAGCTCATACTG
NtANS-qPCR -R	GGAATTAGGCACACACTTTGC
NtUFGT-qPCR -F	GAGTGCATTGGATGCCTTTT
NtUFGT-qPCR -R	CCAGCTCCATTAGGTCCTTG
NtAN2-qPCR –F	GAAGAAAGGTGCATGGACTG
NtAN2-qPCR –R	TCTGCAGCTCTTTCTGCATC
NtAN1a-qPCR-F	ACCATTCTCGAACACCGAAG
NtAN1a-qPCR-R	TGCTAGGGCACAATGTGAAG
NtAN1b-qPCR-F	CTTGAACACTTCTCAAACCGA
NtAN1b-qPCR-R	TGCTAGGGCACAATGTGAAG
NtTubA1-qPCR-F	CTCCTATGCTCCTGTCATTTC
NtTubA1-qPCR-R	GGCGAGGATCACACTTAAC
